# Female Parkinson's disease caregivers have much anxiety and depressive symptom

**DOI:** 10.1002/brb3.787

**Published:** 2017-08-01

**Authors:** Murat Gultekin, Ayten Ekinci, Gozde Erturk, Meral Mirza

**Affiliations:** ^1^ Department of Neurology Faculty of Medicine Erciyes University Kayseri Turkey; ^2^ Department of Biostatistics Faculty of Medicine Erciyes University Kayseri Turkey

**Keywords:** anxiety, caregivers, depression, Parkinson's disease

## Abstract

**Objective:**

Parkinson's Disease Caregivers (PDC) play an important role, especially in the medium and advanced phase of the disease for patients’ daily life activities, treatment, and follow‐up. The aim of this study is to attract attention to the factors which place PDC at risk of psychological problems and to give consideration to these factors.

**Materials and Methods:**

First of all, the 80 participants, who were PDC, filled in the demographic information form. The Hospital Anxiety and Depression Scale (HADS) was applied in order to determine the psychological status of the PDC.

**Results:**

The average age of PDC in the study was found as 47.94. While 11 (13.8%) of the PDC had undergone psychiatric treatment in the past, four of them (5%) were currently receiving treatment. Twenty‐eight (35%) of those who provide care have experience in patient care, whereas 52 (65%) of them have no prior experience in caring for patients. Thirty‐six (45%) of the PDC reported that they had difficulties, which were mostly psychological. According to the HADS which was applied, anxiety was seen in 26 of those who provide care (32.5%), while depression was seen in 41 (51.3%).

**Conclusion:**

This study is the first to provide data on the psychological status of PDC in our country. It is important that PDC's psychological problems are reduced by psychotherapy or, if necessary, by treatment. This situation has a direct negative effect on the patient's daily life activities.

## 1 INTRODUCTION

Those who provide care are a vital part of the patient's life and they serve a useful role in the process of medical treatment. A patient who gets increasingly weak as a result of chronic diseases relies on those who provide care in his/her daily life. Carers help in providing security at home, compliance to treatment, and daily life activities. Furthermore, the patient's response to drugs and treatment is obtained from carers as direct observers and reliable information on the patient's condition is also provided by them. For these reasons, the need to support carers is extremely important for successful treatment management and to safeguard the patient's place in society. However, the role of providing care can create considerable stress (Cifu, Carne, Brown, & Pegg, 2006). According to studies which have been done so far, patients with Parkinson's Disease (PD) and their families think of the disease as significantly destructive. It has been stated that the disease symptoms are related to psycho‐social problems in the patient. It was stated that the situation is seen as bothersome for both patient and carer and is considered as socially shameful (Abudi, Bar‐Tal, Ziv, & Fish, 1997). In addition, the partners of patient with PD were found to have bad social, psychological, and physical profiles (O'Reilly, Finnan, Allwright, Smith, & Ben‐Shlomo, 1996). The age of patients and phase of disease were both found to be significantly related to carers’ psychological problems (Razali, Ahmad, Rahman, Midin, & Sidi, 2011). A very significant relation was found between the depression level of people who care for patients with PD and disease duration. It was stated that the determination of factors which cause depression in Parkinson's Disease Caregivers (PDC) will result in better care (Fernandez, Tabamo, David, & Friedman, 2001). Thus, reducing the psychological problems of PDC by psycho‐training or support groups is important to achieve this (Razali et al., 2011). There are few publications in the literature on the problems related to the care of PDC. As understood from these studies, PD affects both the patient and carer. The anxiety and depression levels of carers of patients with diseases such as cancer, paralytic conditions or paralysis, and dementia in our country were reviewed. However, to the best of our knowledge, we found that no research related to anxiety and depression in PDC has been conducted after the literature review which we performed on the subject. This study is the first to provide data on the psychological status of PDC in our country. Results on this topic were presented after PDC anxiety and depression levels and related factors were determined.

The aim of this study was to attract attention to the factors which place PDC at risk of psychological problems and to give consideration to these factors.

## 2 MATERIAL AND METHODS

### 2.1 Sample

A total of 80 volunteers who were PDC, 47 females and 33 males aged 21–73, for patients with idiopathic PD diagnosis according to the PD diagnostic criteria of the United Kingdom Brain Bank, and who were followed in Erciyes University Medical Faculty's Neurology Polyclinic between January 2016 and April 2016 were included in the study. Exclusion criteria in the study were determined as carers who were under 18 years of age.

This study was approved by Erciyes University Clinical Research Ethics Committee (dated 18.12.2015 and numbered 539). Data were obtained on a volunteer basis after information on the study's goal was provided and written approval was given by the participants.

First of all, a demographic information form was given to participants to fill in. In the next phase, the Hospital Anxiety and Depression Scale (HADS) was applied to determine the psychological status of carers. Information on patients in the demographic information form was obtained from carers with the face‐to‐face interview method. Necessary information for the study related to the clinical status of the patients was added by neurologist. The HADS was completed by participants who were asked to mark the item which was most suitable for their situation. In the event that they were illiterate, questions on the scale were read by a psychologist and the responses of the participants were taken note of; the process took nearly 15 min (10 min for the approval form and 5 min for the demographic information form in the HADS).

### 2.2 The assessment tools used

#### 2.2.1 Demographic information form

A form which was prepared by the researchers was applied for the purpose of determining the age, gender, educational status, and other demographic information belonging to the carers and patients who met the criteria for inclusion in the study. In addition to the demographic information obtained from the form, the degree of relation of the carers to the patient, their circumstances to care for them, patients’ clinical information on PD duration and Hoehn‐Yahr (H&Y) clinical staging were queried and recorded.

#### 2.2.2 Hospital anxiety and depression scale

The scale, which was developed by Zigmond & Snaith (1983), evaluates the levels of anxiety and depression and change. Half of the scale (odd numbers) consisted of items to measure anxiety and the other half (even numbers) consisted of items to measure depression; there were a total of 14 questions. The HADS is a four‐point likert‐type scale in which items are given scores from 0 to 3. The lowest score which can be taken from subscales is 0 and the highest score is 21. In the HADS, 10 was chosen for the cut‐off for the anxiety subscale and 7 for the depression subscale. The validity and reliability of Turkish version of this scale was made by Aydemir, Guvenir, Kuey, & Kulturs (1997).

### 2.3 Statistical analysis

The exact method of the *Pearson's chi‐squared* test statistic was used to compare categorical data in the study. The *Student's t‐test* was used in independent samplings to compare two groups. *Pearson product‐moment correlation analyses* were done to indicate the relations of dependent variances. Data distribution was viewed by means of *Kolmogorov–Smirnov* test statistics. The statistical tests were chosen according to the distribution of data. The analysis of data was reviewed in the SPSS 22 package program. The significance level was accepted as *p > *.05.

## 3 RESULTS

The age range of PDC who participated in the study was found as 21–73 (*M *= 47.94, *SD* = 12.40). The age range of patients was found as 40–87 (*M *= 62.46, *SD* = 10.19). The patients’ H&Y score was between 1 and 4 (*M *= 2.08, *SD* = 0.87).

While 11 (13.8%) of the carers had undergone psychiatric treatment in the past, four of them (5%) were currently receiving treatment. While 28 (35%) of the carers have experience in the care of patients, 52 (65%) of them have no previous experience. Thirty‐six of PDC (45%) reported that they had difficulties which were mostly psychological. The other demographic features of the participants are summarized in Table 1.

**Table 1:** The demographic data of participants

N = 80	Caregivers		Patients with PD	
	*n*	*%*	*n*	*%*
Gender				
Female	47	58.8	39	48.8
Male	33	41.3	41	51.3
Education level				
Not literate	4	5.0	19	23.8
Literate/primary school	39	48.8	41	51.3
Middle/high school	22	27.6	14	17.5
University	15	18.8	6	7.5
Occupation				
Unemployed	39	48.8	40	50.0
Retired	15	18.8	35	43.8
Employee	26	32.5	5	6.3
Relationship				
Spouse	35	43.8		
Daughter/Son	35	43.8		
Brother	3	3.8		
Bride	6	7.5		
Mom/Dad	1	1.3		
Marital status				
Single	8	10.0		
Divorced/widowed	1	1.3		
Married	71	88.8		
Caregiver status				
Sometimes	14	17.5		
Mostly	12	15.0		
Always	54	67.5		
*Mean (Standard deviation)*				
Age	47.94 (±12.40)		62.46 (±10.19)	
Disease duration	—		6.96 (±5.69)	

According to the HADS results, it was seen that 26 of the carers (32.5%) had anxiety and 41 of them (51.3%) had depression. The findings of the HADS are given in Table 2.

**Table 2:** Mean and standard deviation values of hospital anxiety and depression scale scores of the participants

Caregiver	Mean	Standard deviation	*N*
Anxiety	8.03	5.17	80
Depression	8.18	5.82	80

A t‐test was performed for independent groups in order to compare differences in participants’ scores for the HADS by gender. According to the *Kolmogorov–Smirnov* test (*p *>* *.05), data met the normality hypothesis. The results showed that there was a significant difference statistically in anxiety (*t *=* *3.12, *SD* = 78, *p *<* *.01) and depression (*t *=* *3.33, *SD* = 78, *p *<* *.01) scores by gender. The average scores in the anxiety (*M* = 9.38, *SD* = 5.58) and depression (*M* = 9.81, *SD* = 6.14) subscales for females are higher than those for anxiety (*M* = 6.09, *SD* = 3.84) and depression (*M* = 5.85, *SD* = 4.49) for males. The results are given in Table 3.

**Table 3:** *t*‐Test results on the comparison of anxiety and depression scores according to gender

Group	*N*	Mean	Standard deviation	*t*	*SD*	*p*
Anxiety						
Female	47	9.38	5.58	3.12	78	.002^a^
Male	33	6.09	3.84			
Depression						
Female	47	9.81	6.14	3.33	78	.001^a^
Male	33	5.85	4.49			

a *p *<* *.01.

Relations between PDC's age, patients’ age, PD duration, and H&Y score with anxiety and depression were analyzed. When the results of correlation analysis were reviewed, it was found that there was the same direction (positive) and strong significant relation in anxiety and depression scores (*r *=* *.73, *p *<* *.01) and the same direction (positive) and moderate significant relation between H&Y score and patients’ age (*r *=* *.39, *p *<* *.01) and PD duration (*r *=* *.44, *p *<* *.01). Other variable relation with each other was not observed to be statistically significant. The correlation coefficients of variables by analysis results are presented in Table 4.

**Table 4:** The correlation coefficients

		1.	2.	3.	4.	5.	6.
1.	Age of caregiver	—					
2.	Age of patient	.10	—				
3.	H&Y score of patient	.14	.39^a^	—			
4.	Disease duration	.18	.29^a^	.44^a^	—		
5.	Anxiety score	−0.08	.05	.04	.07	—	
6.	Depression score	.09	.10	.21	.21	.73^a^	—

a *p *<* *.01.

Questions which were prepared to assess what areas of the carers’ life were affected by PD were designated as physical area, social area, psychological area, economic area, all of these areas and none of them. Those who said the effect on PDC was physical numbered 6 (7.5%), those who said it was social numbered 1 (1.2%), the number of those who said it was psychological was 36 (45%), the number of those who said it was economic was 3 (3.8%) and the number of those who said that all areas were affected was 16 (20%), the number of those who said that no areas were affected was 18 (22.5%). When participants were asked how they received information on PD, the results were as follows: 49 (61.9%) said that they got information from doctors, 8 (10%) said that they got information from Internet, 4 (5%) said that they got information from television, 4 (5%) said that they got information from books and brochures, 5 (6.2%) said that they got information from patients and patients’ relatives, 10 (12.5%) said that they got information from all these sources.

## 4 DISCUSSION

With this study, the anxiety and depression levels among PDC in our country were provided. Even if our sampling size is not great, it is important in terms of reflecting the first available data. In particular, PD duration, advanced stage of disease (H&Y score) and the age of patients contributed significantly to the risk of PDC developing anxiety and depression. It was shown that these factors have a greater effect on PDC, especially in females.

The comparison results of the anxiety and depression scales by variables which were reviewed in the study are given in Table 5. As a result of the comparison which was conducted, it was found that there was a significant relation between those with anxiety and those without anxiety by gender (*p *=* *.001). Also, was found that there was a significant relation between those with depression and those without depression by gender (*p *=* *.007). The following states were determined: while the rate of anxiety for males was 4 (12.1%), the rate of depression was 11 (33.3%); the rate of anxiety for females was 22 (46.8%), while it was 30 for depression (63.8%). According to other variables, a significant relation between anxiety and depression was not found.

**Table 5:** The comparison of anxiety and depression levels

Gender	Anxiety		Depression		*p* ^*A*^	*p* ^*D*^
	Exist	None	Exist	None		
Female	22 (46.8%)	25 (53.2%)	30 (63.8%)	17 (36.2%)	.001	.007
Male	4 (12.1%)	29 (87.9%)	11 (33.3%)	22 (66.7%)		

*p* ^*A*^, *p* value for anxiety; *p* ^*D*^, *p* value for depression.

It was found in previous studies that there was a direct relation between depression status and the life quality of PDC and care cost (Martin et al., 2007). Eloise et al. evaluated 274 Parkinson’s Disease Caregivers (PDC) in their study. In the study, the effects of PDC personality on their own quality of life were examined. It was shown that depression and anxiety are the greatest factors to affect PDC quality of life. Additionally, while benignancy, awareness, and unorthodoxy manners were seen to have a positive effect, neurotic personalities were found to have a negative effect (Eloise, Sharon, Marilia, & Simon, 2013). PDC with neurotic personalities have difficulties in dealing with stressful situations and they may need to have psychological support.

Schrag et al. evaluated 123 PDC in their study. It was determined that rising depression scores were seen in half of the participants and two thirds of them were affected negatively in their social life. It was shown that the advanced stage of the patient, their psychiatric disease, and frequent falling in patients caused an increase in the burden of carers. In this study, it was determined that PDC age and gender did not contribute to this case in analyses which were done after the disease duration was maintained (Schrag, Hovris, Morley, Quinn, & Jahanshahi, 2006). However, our data show that female PDC, in particular, encountered a greater risk of anxiety and depression. Grun et al. evaluated 53 PDC in their study. It was shown that the nonmotor symptoms of the patient, especially those such as sleep problems and autonomic dysfunction significantly increased the burden of carers. The mood of the carer was found to be related with these factors (Grün, Pieri, Vaillant, & Diederich, 2016).

PDC encounter various risks in terms of their psychological health. This has been revealed in many studies in the past (Lökk, 2009; McLaughlin et al., 2010). In particular, individuals’ personality, social support, and training have a determining role in this issue. Both patients’ and carers’ quality of life and the success of treatment are related directly to these factors. In this study, nearly 61% of PDC stated that they received information on PD from doctors. Thus, adequate time should be given to individuals who are PDC by health professionals and wide information sources should be provided. To protect physical and psychological health, awareness should be increased and social support groups should be formed for PDC (Simons, Thompson, & Smith Pasqualini, 2006).

## 5 CONCLUSION

The data in this study show that, in particular, female PDC in our country are at higher risk in terms of anxiety and depression. There is a need for multicentered and wide‐scale studies to verify the data and to determine the risk factors detailed in our study.

## CONFLICT OF INTEREST

There is no financial, professional, and personal relationships with the potential to bias the work.

## AUTHOR CONTRIBUTIONS

Substantial contributions to conception and design of, or acquisition of data or analysis and interpretation of data: MG, AE, GE. Drafting the article or revising it critically for important intellectual content: MG, MM. Final approval of the version to be published: MG, MM.
